# Impact of peripheral muscle strength on prognosis after extubation and functional outcomes in critically ill patients: a feasibility study

**DOI:** 10.1038/s41598-021-95647-7

**Published:** 2021-08-09

**Authors:** Tsung-Hsien Wang, Chin-Pyng Wu, Li-Ying Wang

**Affiliations:** 1 School and Graduate Institute of Physical Therapy, College of Medicine, National Taiwan University, Taipei, Taiwan; 2 Departments of Critical Care Medicine, Landseed International Hospital, Taoyuan, Taiwan

**Keywords:** Health care, Medical research, Risk factors

## Abstract

The influence of peripheral muscle strength on prognosis after extubation and subsequent functional outcomes is not evident. The objectives of this study were to determine (1) whether peripheral muscle strength can be used as a predictor for patients’ prognoses after extubation, and (2) whether the peripheral muscle strength before extubation is correlated with patients’ subsequent ambulation ability and in-hospital mortality. This study was a prospective observational cohort study. A hand-held dynamometer was used for evaluated the muscle strength of the biceps and quadriceps right before extubation. Besides, after the patients had been transferred from the ICU to the general ward, a 2-minute walk test was performed. A total of 52 patients were enrolled in this study, and the rate of extubation failure was 15%. The muscle strength of the quadriceps was significantly correlated with the prognosis after extubation, 48% of the patients were able to ambulate after being transferred to the general ward. The overall mortality rate was 11%, and there was a significant correlation between the biceps muscle strength and in-hospital mortality. Peripheral muscle strength may serve as an important predictor of a patients’ prognoses after extubation. Poor peripheral muscle strength is indicative of not only a higher risk of re-intubation but also higher in-hospital mortality and poorer functional outcomes.

Trial registration: ISRCTN16370134. Registered 30 May 2019, prospectively registered. https://www.isrctn.com/ISRCTN16370134.

## Introduction

Critically ill patients often require the use of ventilators to maintain their lives. Endotracheal intubation and ventilation are usually needed for the management of respiratory failure, heart failure, or respiratory support after surgery^1^. Although ventilators can temporarily help maintain the vital signs of patients, they may also cause complications, and muscle weakness is one of the most common ones. Muscle weakness commonly seen in the ICU is known as ICU-acquired weakness and is usually related to poor prognosis^2–4^. Some studies revealed that weakness after a severe illness may lead to an increased number of days on a ventilator^5^, higher risk of relapse requiring intensive care^6^, higher mortality^7^, and a higher rate of extubation failure^8^.

Determining the optimal time to discontinue mechanical ventilation is a critical issue because if the extubation fails and leads to re-intubation, patients might have a higher mortality risk^9,10^. Therefore, daily assessment of the patients’ readiness to be weaned from the ventilator and extubated is based on the daily spontaneous breathing trial (SBT) results. Although patients are extubated only when they have successfully passed SBT and other indicators for weaning from the ventilator also satisfy the criteria for extubation, some studies pointed out that 10–23% of the patients will still experience extubation failure and will have to be re-intubated to use the ventilator within 48–72 h^9,11–15^.

Even if the patients could smoothly wean from the ventilator and be transferred out of the ICU, 1/3rd of them would still face severe dysfunction, and 1/4th of them would have reduced mobility^16,17^. Few data are available on whether muscle weakness, a complication of patients in the ICU, is correlated with the prognosis of subsequent functional activities^18,19^. Moreover, these studies are usually investigated on long-term prognosis, while there are still no data related to the post-acute care functional prognosis.

Although many studies have shown that ICU-acquired weakness would make it difficult for patients to wean from the ventilator, studies investigating the correlation between peripheral muscle strength and extubation outcome remained limited. In addition to the lack of relevant research on the correlation between peripheral muscle strength and prognosis after extubation, as far as we know, there is no investigation on patients’ prognosis for post-acute care functional activity so far. Therefore, in this feasibility study preliminarily explored whether peripheral muscle strength is correlated with extubation outcomes, the subsequent functional outcome and prognosis.

Our study aimed to measure the muscle strength of the biceps and quadriceps using the hand-held dynamometer. The main objective was to explore the correlation between the biceps and quadriceps muscle strength and patients’ extubation outcome. We hypothesized that this detection method would provide a rapid and non-invasive measurement approach for the evaluation of timely extubation. Furthermore, this study aimed to investigate whether the muscle strength of the biceps and quadriceps was correlated with patients’ post-acute care ambulation function and hospital mortality.

## Materials and methods

This study is a prospective observational cohort study conducted from January 2019 to January 2020. The inclusion criteria for the subjects were as follows: age ≥ 20 years, having been ventilated for > 48 h in the Landseed International Hospital, having undergone general surgery or trauma surgery, or receiving internal medicine care. Patients were excluded if they were diagnosed with brain death; if they were ventilator-dependent or tracheostomy; if they could not perform the muscle strength test (due to other pre-existing conditions, e.g., amputation, musculoskeletal diseases, and cachexia); if they showed neuropsychiatric symptoms, which led to the inability to comply with the instructions; if they were an adult with challenged mental capacity.

**Figure 1:**
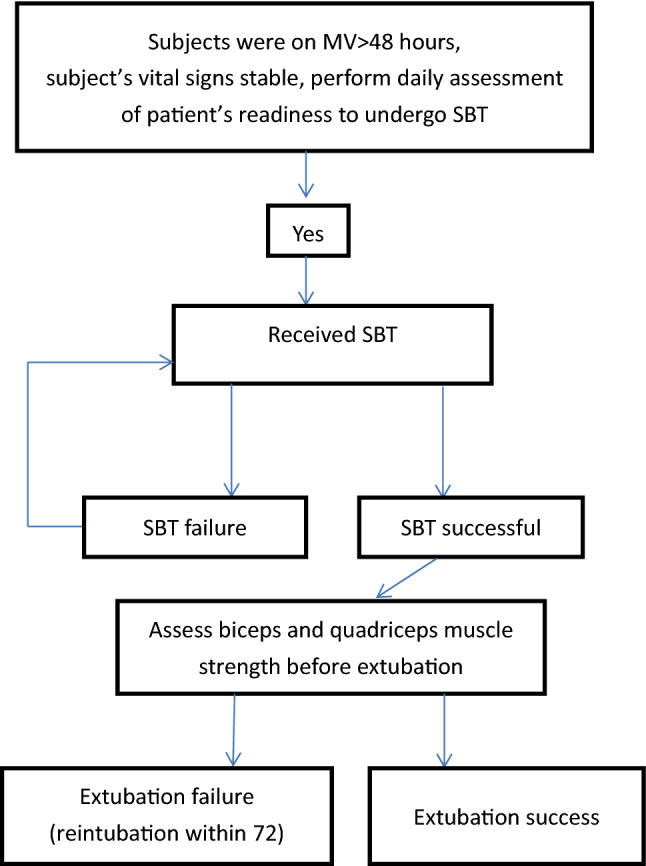
Study flowchart. *SBT* spontaneous-breathing trial, *MV* mechanical ventilation.

The study was performed in accordance with current ethical guidelines (Declaration of Helsinki) and we follow all methods were performed in accordance with the relevant guidelines and regulations. Ethics approval was obtained from the Landseed International Hospital ethics institutional research committee (19–017-B1). All patients or their designated relatives provided informed consent (Fig. 1).

Once a patient’s vital signs were stable and the disease conditions were under control, the attending physician would decide when to allow the patient to start the spontaneous breathing trial based on the weaning protocol of the Landseed International Hospital. Once the patient had stable vital signs and unimpaired consciousness, the physiotherapist would start to test the muscle strength of the biceps and quadriceps. The test was conducted on the day of the patient’s extubation, with the data measured right before extubation. The patient’s dominant hand and dominant foot were used as the test sites. The test was performed three times for each patient at an interval of 30 s, and the recorded attempt was the highest strength result.

### Breathing trial

The patients would undergo the spontaneous breathing trial (SBT) before extubation, which tested whether they could breathe on their own with a T-piece for 60 min. A patient’s spontaneous breathing trial would be terminated if any of the following situations would occur during the trial: respiratory rate > 35 bpm, blood oxygen saturation < 90%, heart rate > 140 bpm or presenting a change > 20%, systolic blood pressure < 90 mmHg or > 180 mmHg, anxiety or restlessness, diaphoresis, respiratory distress, angina pectoris, or a patient complaining of breathing difficulty. If the test was successful, the attending physician would assess the patient again and decide whether to proceed to extubation. In our study, the routine clinical procedures before extubation followed the medical care procedures of the hospital. The only difference was the addition of a muscle strength test of the biceps and quadriceps, measured by a physiotherapist, within 1 h before extubation.

Extubation failure: Extubation failure was defined as the need to re-intubate a patient within 72 h after the patient had been on the ventilator for > 48 h, passed the spontaneous breathing trial, and was extubated.

### Clinical assessment of muscle strength

A physiotherapist used microFET to evaluate the muscle strength of the biceps and quadriceps during isometric contraction. The test method was based on the test criteria for the biceps and quadriceps established by the National Isometric Muscle Strength (NIMS) Database Consortium.

The biceps test method procedure: the patient was in the semi-Fowler position, with the elbow bent at 90º and the hand in supination. The patient was asked to perform elbow bending; the tester applied the resistance of the test instrument to the patient’s wrist, asked the patient to counteract the resistance with maximum strength for 3 s, and recorded the maximum resistance. The patient was asked to repeat it three times, and the attempt with the highest measurement was recorded. The quadriceps test procedure: The patient was in the semi-Fowler position, with their knee extended; the tester applied the resistance of the test instrument to the front of the lower extremity near the ankle, asking the patient to counteract the resistance with maximum strength for 3 s, and recorded the maximum resistance. The patient was asked to repeat it three times, and the attempt with the highest score was used.

### Collection, processing, evaluation, and statistical analysis of research data

In our study, we enrolled patients aged > 20 years, ventilated for > 48 h, and meeting the inclusion criteria. The data collected included the patients’ main diagnosis, age, sex, APACHE II score (acute physiology score + age points + chronic health points), consciousness level was evaluated using the estimated Glasgow Coma Score (eGCS)^20^, number of days on a ventilator, number of ICU days, muscle strength score, maximum inspiratory pressure (MIP), rapid shallow breathing index (RSBI), success or failure of extubation, and survival or death. Since it is not possible to assess verbal response in intubated patients, in this research we used the estimated GCS, which estimates the verbal score based on the GCS eye opening and motor scores. Muscle strength was measured only before the first extubation and would not be measured for any subsequent extubation. However, data such as the number of ICU days and days on a ventilator would continue to be collected until the patient was transferred out of the ICU.

### Evaluation of the patients’ functional prognosis

#### Procedure: 2-minute walk test

After a patient was transferred out of the ICU, the physiotherapist would record the patient’s first time to get out of bed to walk and evaluate the patient’s 2-minute walk test distance. The 2-minute walk test would be carried out according to the recommendations of the American Thoracic Society^21^ in a space with a length of more than 50 ft (15.2 m), and the physiotherapist would instruct the patient to walk as much as possible within the 2 min until told to stop. The patient could slow down or rest during the test if he/she wanted to, and the pace was determined solely by the patient. When there was 1 min left, the patient would be informed: “You are doing well. You have 1 min left.” At 2 min, the patient would be asked to stop, and his/her walking distance would be recorded.

### Outcome

The primary outcome was extubation failure. Secondary outcome measures were post-acute walking ability, ICU and hospital mortality; whether peripheral strength were associated with the walking ability and risk of mortality.

### Statistical analyses

Data were expressed as mean and standard deviation. T-test was used to examine whether there were statistically significant differences between the two groups in continuous variables such as age, eGCS score, disease severity score, number of days on a ventilator, number of ICU days, rapid RSBI and muscle strength score, MIP, and the 2-minute walk test distance. Chi-square test (χ^2^ test) was used to test whether there were statistically significant differences in categorical variables such as sex, whether re-intubation was performed, whether the patient had died, and whether the patient had been re-admitted to the ICU. Finally, univariate logistic regression was used to analyze the correlation factors of extubation failure and mortality. Univariate and multivariate logistic regression were used to analyze the correlation factors of walking ability. All the analyses were carried out using SPSS Version 17.0.

### Ethics approval and consent to participate

Ethics approval was obtained from the Landseed International Hospital ethics institutional research committee (19-017-B1). All patients or their relatives agreed and signed an informed consent.

### Consent for publication

The authors give consent for publication.

## Results

During the period of the study, 52 people satisfied the inclusion criteria, and their demographic data are shown in Table 1. The average age of all patients was 69 years. Concerning sex, males accounted for approximately 60% and females accounted for approximately 40% of the participants. Concerning disease severity, the average APACHE II score on admission was 22 points. Concerning the primary diagnosis for admission, 60% were respiratory diseases, followed by 15% cardiac diseases and 12% gastrointestinal diseases. The main cause of intubation was respiratory failure, accounting for 65% of intubations, followed by shock, accounting for 17%. In terms of comorbidity, hypertension accounted for up to 50% of the comorbidities. There was no significant difference in diagnosis, the reason for intubation, and comorbidity between the two groups (the extubation success group and the extubation failure group).

**Table 1:** Characteristics of the patients at admission.

Characteristics	Total (n = 52)	Extubation successful (n = 44)	Extubation failure (n = 8)	*P* value
Age, y	69.4 ± 14.0	68.8 ± 13.8	72.9 ± 15.7	0.45
Sex, male (%)	31 (60)	25 (57)	6 (75)	0.34
APACHE II on ICU admission	21.8 ± 9.2	20.9 ± 8.1	26.3 ± 13.9	0.33
BMI (kg/m^2^)	23.6 ± 4.7	23.7 ± 4.8	23.4 ± 4.4	0.88
**ICU diagnosis on ICU admission (%)**				0.35
Heart disease	8 (15)	7 (16)	1 (13)	
Respiratory insuffucuency	31 (60)	26 (59)	5 (63)	
Gastroenterology	6 (12)	5 (11)	1 (13)	
Renal disease	2 (4)	2 (5)	0 (0)	
Hemodynamic insuffiency	3 (6)	3 (7)	0 (0)	
Trauma	1 (2)	0 (0)	1 (13)	
Other	1 (2)	1 (2)	0 (0)	
**Main reason for intubation (%)**				0.24
Acute respiratory failure	34 (65)	31 (70)	3 (38)	
Shock	9 (17)	7 (16)	2 (25)	
Surgery	7 (13)	5 (11)	2 (25)	
Cardiac arrest	2 (4)	1 (2)	1 (13)	
**Comorbidities (%)**				
Chronic respiratory disease	12 (23)	8 (18)	4 (50)	0.07
Chronic cardiac disease	15 (29)	12 (27)	3 (38)	0.68
Diabetes	18 (35)	16 (36)	2 (25)	0.70
Hypertension	26 (50)	21 (48)	5 (63)	0.70

*APACHE II* acute physiology and chronic health evaluation II, *BMI* body mass index, *ICU* intensive care unit.

The data measured on the same day right before extubation are shown in Table 2. The re-intubation rate was 15.4%. The average muscle strength of the biceps and quadriceps was 15 pounds and 17 pounds, respectively. The mean muscle strength of the quadriceps in the extubation success group was 18 pounds. In comparison, in the extubation failure group, it was 11 pounds, with a statistically significant difference between the two groups. There were no statistically significant differences in other common weaning indicators such as RSBI, minute ventilation (VE), and MIP.

**Table 2:** Patient characteristics at time before planned extubation.

Characteristics	Total (n = 52)	Extubation successful (n = 44)	Extubation failure (n = 8)	*P* value
Lactate (mmol/L)	1.4 ± 0.4	1.4 ± 0.5	1.3 ± 0.3	0.69
RSBI	67.1 ± 30.7	65.7 ± 29.0	75.1 ± 39.9	0.43
Estimated Glasgow Coma Scale	14.1 ± 0.7	14.0 ± 0.7	14.2 ± 0.7	0.53
pH	7.46 ± 0.05	7.46 ± 0.05	7.44 ± 0.05	0.37
PaCO_2_ (mmHg)	41.9 ± 7.5	42.1 ± 6.7	41.6 ± 11.7	0.91
PaO_2_ (mmHg)	89.5 ± 18.3	90.3 ± 17.9	85.5 ± 21.4	0.51
HCO^3−^ (mmol/L)	28.9 ± 4.0	29.2 ± 3.6	27.5 ± 5.9	0.43
PaO_2_/FIO_2_ (mmHg)	287.5 ± 71.4	288.4 ± 70.6	282.9 ± 80.1	0.85
Minute ventilation (L/min)	7.7 ± 2.8	7.6 ± 2.8	8.2 ± 2.6	0.53
Compliance (mL/cmH_2_O)	53.3 ± 21.2	53.3 ± 20.9	53.4 ± 24.2	0.99
Resistance (cmH_2_O˙s/L)	13.7 ± 4.7	14.1 ± 4.9	11.5 ± 3.0	0.16
SpO_2_ (%)	97.2 ± 2.2	97.5 ± 1.9	95.6 ± 3.4	0.18
Respiratory rate (per minute)	16.9 ± 4.3	16.8 ± 4.5	17.0 ± 3.4	0.93
Heart rate (per minute)	87.7 ± 13.5	87.9 ± 13.5	86.4 ± 14.5	0.76
MIP (cmH_2_O)	38.7 ± 10.4	39.1 ± 11.1	36.0 ± 4.1	0.44
Biceps (pound-force)	15.2 ± 5.7	15.8 ± 5.6	12.1 ± 5.0	0.09
Quadriceps (pound-force)	17.0 ± 7.8	18.1 ± 7.7	11.3 ± 6.0	**0.02**

*RSBI* rapid shallow breathing index, *MIP* maximum inspiratory pressure. Bold values are statistically significant.

The patients’ prognoses are shown in Supplemental Table 1 (see Additional file 1). The average number of days on a ventilator, average number of ICU days, and the mortality rate of all patients were 8 days, 12 days, and 11%, respectively. The average days on a ventilator (6.8 vs. 16.4; P = 0.017) and average days of ICU stay (10.3 vs. 22.2; P = 0.003) were significantly lower in the extubation success group than in the extubation failure group. Further analysis showed that the APACHE II score was positively correlated with the number of days on a ventilator and the number of ICU days [Supplemental Table 2 (see Additional file 2)], with statistically significant differences (P = 0.03).

### Univariate logistic regression analysis

According to the univariate (Table 3) logistic regression analysis models, longer duration of MV and ICU stays are predictor of extubation failure. Besides that, it was found that the muscle strength of the quadriceps was significantly correlated with the extubation outcomes, and the less the muscle strength of the quadriceps, the higher the risk of re-intubation.

**Table 3:** Univariate logistic regression analysis of factors associated with extubation failure within the first 3 days following planned extubation. Bold values are statistically significant.

Variables	Beta coefficient	S.E.	Odds ratio (95% CI)	*P* value
Biceps	−0.14	0.08	0.87 (0.74–1.03)	0.10
Quadriceps	−0.21	0.10	0.81 (0.66–0.98)	**0.038**
MIP	−0.002	0.01	1.00 (0.99–1.01)	0.75
RSBI	0.01	0.01	1.01 (0.99–1.04)	0.42
Minute ventilation	0.08	0.13	1.09 (0.84–1.41)	0.53
Compliance	0.00	0.02	1.00 (0.97–1.04)	0.99
Resistance	−0.17	0.12	0.85 (0.67–1.07)	0.16
APACHE II	0.06	0.04	1.06 (0.98–1.15)	0.15
Age	0.02	0.03	1.02 (0.97–1.09)	0.44
BMI	−0.01	0.08	0.99 (0.84–1.16)	0.87
Duration of MV	0.34	0.12	1.40 (1.11–1.76)	**0.004**
ICU stays	0.47	0.17	1.61 (1.15–2.24)	**0.006**

### Functional outcomes

Approximately 48% of the previously intubated patients could walk in the general ward after weaning from the ventilator and transferring to the general ward. The average time it took them to get out of bed to walk for the first time was 3 days after transferring to the general ward. The average distance of the 2-minute walk test was approximately 38 m [Supplemental Table 3 (see Additional file 3)]. Further analysis showed that the muscle strength of the quadriceps measured before extubation was correlated with the patients’ functional activity (getting out of bed and walking), with statistically significant difference (21.1 vs. 13.2; P = 0.001), and the better the muscle strength of the quadriceps before extubation, the higher the chance of being able to walk after transferring to the general ward [Supplemental Table 4 (see Additional file 4)].

**Table 4:** Multivariate logistic regression for regaining ambulation ability of potential predictor after ICU discharge. Bold values are statistically significant.

Variables	Beta coefficient	S.E.	Odds ratio (95% CI)	*P* value	
Age	−0.11	0.50	0.89 (0.81–0.98)		**0.02**
ICU stays	−0.30	0.11	0.74 (0.59–0.92)		**0.01**
Biceps	−0.22	0.11	0.81 (0.65–1.01)		0.06
Quadriceps	0.24	0.09	1.27 (1.07–1.50)		**0.01**
MIP	−0.01	0.01	0.99 (0.98–1.01)		0.47

### Regaining ambulation ability of potential predictor

Multivariate logistic regression analysis was used to analyze the factors that potentially influenced the patients’ ambulation ability after being transferred out of the ICU. It was found that the age of the patient was positively associated with the number of ICU days and negatively associated with quadriceps muscle strength. These three factors would lead to a higher probability that a patient would not, subsequently, be able to walk (Table 4).

### Mortality

In our study, we established a mortality rate of approximately 11%. Further investigation revealed that the muscle strength of the biceps measured before extubation was correlated with the patient probability of in-hospital mortality with a statistically significant difference [Supplemental Table 5 (see Additional file 5)]. The biceps muscle strength of the patients who died during hospitalization was significantly less than that of the survivors (11.0 vs. 15.8; P = 0.044). Furthermore, univariate logistic regression was used to analyze the risk factors for the in-hospital mortality, and it was found that the muscle strength of biceps and BMI were negatively associated with the risk of in-hospital mortality (Table 5).

**Table 5:** Univariate logistic regression analysis of In-hospital mortality. Bold values are statistically significant.

Variables	Beta coefficient	S.E.	Odds ratio (95% CI)	*P *value
Biceps	−0.26	0.13	0.77 (0.59–0.99)	**0.048**
Quadriceps	−0.05	0.07	0.95 (0.83–1.08)	0.44
MIP	−0.01	0.04	0.99 (0.92–1.07)	0.80
RSBI	0.02	0.01	1.02 (0.99–1.05)	0.20
Minute ventilation	0.10	0.15	1.10 (0.83–1.47)	0.50
Compliance	−0.03	0.03	0.97 (0.93–1.02)	0.28
Resistance	−0.01	0.09	0.99 (0.82–1.19)	0.91
APACHE II	0.03	0.04	1.03 (0.95–1.12)	0.50
Age	0.00	0.03	1.00 (0.94–1.06)	0.99
BMI	−0.25	0.13	0.78 (0.61–0.99)	**0.045**
Duration of MV	−0.08	0.11	0.92 (0.74–1.15)	0.47
ICU stays	0.04	0.06	1.04 (0.93–1.17)	0.49

## Discussion

### Main findings

The results of this study suggest that poor muscle strength of quadriceps is an important predictor for extubation failure in ICU patients on a ventilator. Patients with poorer muscle strength of quadriceps would have a significantly higher risk of re-intubation.

Re-intubation is a significant clinical event for patients in the ICU. It increases the risk of infections, respiratory tract injuries, and respiratory muscle weakness. It may also reduce the patients’ subsequent functional performance and quality of life^1,22^, and increase the hospitalization days and mortality in the ICU^1,12,23^. Some studies found that the mortality rate of re-intubated patients increased from 25 to 50%^10,23,24^. Therefore, it is essential to carry out an accurate assessment for extubation. There are many related factors for extubation prediction, among which coughing ability and ICU-acquired weakness are significant independent factors^10,25,26^. Coughing function is positively correlated with the strength of respiratory muscles, and maximum inspiratory pressure (MIP) and maximum expiratory pressure (MEP) are often used as its related indexes. Coughing ability is a significant predictor^26,27^, some studies pointed out that if patients do have effective coughing function at the time of extubation, it will lead to a higher risk of re-intubation^28–32^. Patients cannot cough effectively because of the changes in the functions of expiratory muscles, including the diaphragm, accessory muscles of respiration, and abdominal muscles^33,34^. In addition to systemic muscle weakness, the throat muscles will be affected, causing patients to have dysphagia^35^. The mechanism of cough is mainly the contraction of expiratory muscles, particularly the abdominal muscles^33,34^. Covalcante et al.^36^ investigate the relationship of expiratory muscle strength with the spontaneous breathing of individuals on mechanical ventilation. This is a cross-sectional study with participants aged between 18 and 79 years, they found that better abdominal muscle was associate to better values in RSBI. Coughing ability is significantly correlated with peripheral muscle strength; thus, weakness in coughing is less common in a patient with normal peripheral muscle strength^8^. The quadriceps overlap with some abdominal muscles and the weakness of the quadriceps may affect the strength of the expiratory muscles; this may explain the fact that those with poor quadriceps muscle strength also have poor coughing function, thus increasing their risk of re-intubation. All previous studies investigated the peripheral muscle weakness leading to the patients’ difficulty in weaning from the ventilator and extending the number of days on a ventilator^5,37–39^. However, there are very few studies that explore the relationship between peripheral muscle strength on the prognosis after extubation. Two studies tried to examine the prognosis after extubation using grip strength^40,41^. Their results showed that patients with poorer grip strength would spend more days on a ventilator and have a higher risk of difficulty in weaning from it. The grip strength results were not significantly correlated with the prognosis after extubation in either of the studies. Two other studies used the Medical Research Council (MRC) scale to evaluate peripheral muscle weakness and explore whether the results were correlated with the prognosis after extubation^8,42^. Their results showed that patients with lower MRC scores had a higher risk of re-intubation.

To the best of our knowledge, our study is the first to use this testing method of peripheral muscle strength for the evaluation of patients before extubation to determine if it is an important predictor of prognosis after extubation. Our study results confirmed that the muscle strength of the quadriceps is indeed an important predictor of patient prognosis after extubation. There are many reasons for extubation failure. ICU-acquired weakness is a common complication of patients’ stay in the ICU and may be accompanied by muscle and nerve problems at the same time. Previous studies have showed that more than 50% of ventilated patients would have this complication^43,44^. Muscle weakness caused by this complication is often systemic and symmetrical, and it results in not only peripheral muscle weakness but also respiratory muscle weakness^45^, thereby causing the extension of patients’ time on a ventilator^37,38^. Therefore, the ICU-acquired weakness may be an essential risk factor for extubation failure. A recent study has showed that common causes of muscle weakness include old age, septicemia, electrolyte imbalance, steroid therapy, use of neuromuscular blockers, bed confinement, and overdosage of sedatives^46^. In our study, other factor such as APACHE II score (r = − 0.28, P = 0.046) and age (r = − 0.42, P = 0.002) correlated negatively with the biceps, and only age (r = − 0.46, P = 0.001) correlated negatively with the quadriceps muscle strength. In extubation failure group, half of the patient with chronic respiratory diseases and higher RSBI. The average APACHE II score on admission was 22 points, and extubation failure group appears sicker as indicated by higher APACHE II scores. While the difference in APACHE II scores between groups did not reach statistical significance, there was a trend towards higher scores in extubation failure group, suggesting that the sample may be underpowered. Even absence of statistical significance, it requires more research to verify the correlation.

In the past, there were many criteria for extubation, and there were some patients who passed the breathing trial and met those criteria. However, other patients had extubation failure. We hope that our study provides some new parameters of evaluation and thus improves the extubation success rate.

Regarding the functional prognosis evaluation, to the best of our knowledge, this is the first study to evaluate the post-acute care functional prognosis of ventilated ICU patients continuously after extubation. This study found that about 48% of the patients could get out of bed to walk after being transferred out of the ICU and before discharge from the hospital. On average, 3 days was the average time it took patients to ambulate after leaving the ICU. Besides, this study also found that the muscle strength of the quadriceps before extubation was significantly correlated with the patient ambulation ability. The muscle strength of the quadriceps was negatively associated with the risk of ambulation inability in the post-acute care period. In this study, multivariate logistic regression analysis showed that in addition to the muscle strength of the quadriceps, the age and the number of hospitalization days were important factors that affected the subsequent recovery of the ambulation ability of patients. We found that an older age and an extended stay in the ICU presented a higher risk of being unable to walk in the post-acute care period.

The walk test has been widely used as an essential index to evaluate patients’ functional exercise capacity. The American Thoracic Society originally suggested the 6-minute walk test. However, many patients could not complete the test for such a long time; thus, a 2-minute walk test was to be used instead^47^. The 2-minute walk test has excellent reliability and validity for patients with different diseases^48–50^, and its results are significantly correlated with those of the 6-minute walk test^51,52^. Therefore, the 2-minute walk test was used in this study to evaluate the prognosis of patients’ functional activities. The average 2-minute walk test distance of patients in this study was approximately 38 m. To our knowledge, no data have been published on the 2-minute walk test distance of patients who have been evaluated after extubation upon their transfer out of the ICU.

Ambulation ability has been widely used to evaluate the functional prognosis of patients. Selman et al.^53^ studied the 2-minute walk test in a group of healthy people. A total of 390 people were enrolled, with an average age of 52 years (range, 36–68 years), and the average distance of the 2-minute walk test was 211 m. Butland et al.^52^ investigated the 2-minute walk test distance in patients with chronic obstructive pulmonary disease. They enrolled 10 patients with an average age of 61 years, and the average 2-minute walk test distance was 149 m. Scalzitti et al.^54^ studied the 2-minute walk test distance in a group of patients with multiple sclerosis. The number of cases enrolled was 28, with an average age of 51 years. The average 2-minute walk test distance was 115 m. The 2-minute walk test distance in the population of our study was significantly less than that of other study populations. The overall cardiopulmonary function of patients declines after experiencing respiratory failure. In addition to their walking ability, functional endurance and overall life functioning is affected, and other studies show similar results^1,55,56^. Therefore, identifying the risk factors related to limiting ambulation could allow timely prevention of ICU-acquired weakness and reduction of its sequelae.

The last important finding of our study is that the biceps muscle strength is an important predictor of in-hospital mortality for patients on ventilator in the ICU. Biceps muscle strength and BMI were positively associated with survival rate. Other factors, such as age and APACHE II, were not correlated with in-hospital mortality. In this study, age was not predictors of in-hospital mortality. Another study by Wang et al.^57^ also revealed that age was not correlated with in-hospital mortality. However, other studies have shown that age could be a predictor of in-hospital mortality. Older patients in the ICU generally have more health problems and more significant physiological function damage; hence, higher mortality may be expected than that in the general ward. On the contrary, Weijs et al.^58^ pointed out that BMI is not an independent predictor of in-hospital mortality. However, other studies reported BMI as a predictor of that mortality^57,59,60^. Peripheral muscle strength is an important predictor of in-hospital mortality; our study findings confirmed that the biceps muscle strength is an independent predictor of mortality. Numerous other studies also report similar conclusions^3,57,61,62^. Skeletal muscles have an important influence on the glucose and protein metabolism of the body, as well as in the maintenance of the nutritional homeostasis and the supply of nutrients to the immune system to repair damaged tissues and manage the inflammatory response^63^. Therefore, if the muscle strength is significantly less, it may be due to excessive muscle consumption as a result of a disease, thus affecting the body’s ability to recover and provide an inflammatory response to external factors, resulting in decreasing the survival rate of the patients. Peripheral muscle strength may therefore be considered as an important indicator of patients’ general health condition.

### Strength and limitations of the study

The advantage of this study is that it evaluates, for the first time, the patient prognosis after extubation through measurement of both the peripheral muscle strength right before extubation and the patients’ ambulation ability in the post-acute care phase. Additionally, it further analyzed the related factors that affect patients’ subsequent walking ability. Our study identified the risk factors for re-intubation and impaired ambulation. The study results could be applied as critical points for the evaluation of patients in the ICU in the future.

There are some limitations to this study. First, the number of samples was small, and considering that this was a single-center study, there might be a possibility of sample selection deviation, and the extrapolability of the results may be limited. Large-scale investigations are needed to confirm the results of this study in the future. Second, our research did not use electromyography or ultrasonography to evaluate the diaphragm function. However, we used the MIP as a diaphragm assessment. As pointed out by previous studies, patients who developed peripheral muscle weakness in the ICU also had a higher rate of diaphragm weakness^37,38^, thus increasing the risk of extubation failure. Therefore, our study provides a quick and simple way to evaluate peripheral muscle strength, establishing the strength of the quadriceps as an important predictor of the prognosis after extubation, and thus promoting the peripheral muscle strength test as an excellent method to evaluate extubation risk even without functional evaluation of the diaphragm. Finally, because this study required patients’ cooperation in the tests, even though sedatives were discontinued for the patients during the test, incomplete metabolism of the drugs might have still influenced the results. However, compared with the previous use of the MRC scale in the muscle strength evaluation of patients, our study testing method proves to be much simpler, while minimizing the influence of patients’ hope for the future and possible drug effects on the muscle strength evaluation, and finally presenting it as a more suitable option for clinical application.

## Conclusions

This intervention is feasible and appears to be associated with improvement in clinical outcomes, and that larger studies are needed to confirm predictive power of peripheral strength. Our study determined that poor peripheral muscle strength would lead to a higher risk of extubation failure. Besides, peripheral muscle strength before extubation is also significantly correlated with the ambulation ability of patients in the post-acute care period and in-hospital mortality. Our results emphasize the importance of peripheral muscle strength for patients on ventilator in the ICU. Progressive muscle weakness presents a severe impact on prognosis. Therefore, timely detection and treatment could help prevent ICU-acquired weakness. Future research in this direction could help establish the peripheral muscle strength threshold for ventilator weaning and add new extubation criteria, thus providing patients with a higher probability of successful extubation, reducing the serious sequelae caused by re-intubation, and detecting in advance a higher mortality risk in patients confined to bed.

## Supplementary Information

Supplementary Tables.

## Data Availability

All data are accrued from the literature.
