# Supplementary material for: Impact of peripheral muscle strength on prognosis after extubation and functional outcomes in critically ill patients: a feasibility study
Source: Sci Rep. 2021 Aug 9;11:16082. doi: 10.1038/s41598-021-95647-7 (PMC8352971; doi:10.1038/s41598-021-95647-7)
Supplement: Supplementary file 1 — Supplementary Tables. [file 41598_2021_95647_MOESM1_ESM.pdf]

Additional file 1.

Supplemental Table 1. Clinical outcomes of all patients

Characteristics	Total (n= 52)	Extubation successful (n= 44)	Extubation failure (n= 8)	*P* value
**Duration of MV, d**	8.3 ± 5.7	6.8 ± 3.4	16.4 ± 8.7	**0.017**
**ICU stays, d**	12.2 ± 6.4	10.3 ± 4.0	22.3 ± 7.8	**0.003**
In-Hospital mortality, n (%)	6 (11.5)	5 (11.4)	1 (12.5)	0.93

Additional file 2.

Supplemental Table 2. Correlation between Duration of MV and ICU stays, related variables

Variables	Duration of MV		ICU stays	
	r	*p*	r	*p*
Age	-0.17	0.23	-0.15	0.30
**APACHE II**	0.31	**0.03**	0.30	**0.03**
Minute ventilation	0.26	0.06	0.23	0.11
RSBI	-0.11	0.44	-0.01	0.94
Compliance	0.09	0.53	-0.01	0.96
Resistance	-0.18	0.21	-0.21	0.13
PaO2/FIO2	-0.08	0.59	-0.07	0.64
Lactate	0.02	0.89	0.00	0.99
MIP	-0.01	0.96	0.06	0.65
Biceps	-0.07	0.61	-0.16	0.25
Quadriceps	-0.13	0.37	-0.19	0.18
BMI	0.03	0.84	-0.06	0.70

Additional file 3.

Supplemental Table 3. Functional progress following ICU admission, patient's pre-admission functional level were independent ambulation.

Ambulation in ward (n=25/52)	1st day to ambulation (D)	2 MWD (m)
48.1%	3.1 ± 2.5	37.7 ± 32.5

Additional file 4.

Supplemental Table 4. The relationship between muscle strength and ambulation ability

Characteristics	Total	Ambulation in ward		*P* value
	(n= 52)	Yes (n= 25)	No (n= 27)	
Biceps	15.2 ± 5.7	16.8 ± 6.6	13.8 ± 4.3	0.06
**Quadriceps**	17.0 ± 7.8	21.1 ± 8.0	13.2 ± 5.4	**0.001**

Additional file 5.

Supplemental Table 5. The relationship between Muscle strength and In-hospital mortality

Characteristics	Total (n= 52)	Survivors (n= 46)	Non-Survivors (n= 6)	*P* value
**Biceps**	15.2 ± 5.7	15.8 ± 5.6	11.0 ± 4.2	**0.044**
Quadriceps	17.0 ± 7.8	17.3 ± 7.9	14.7 ± 7.1	0.51
